# Tolerability and efficacy of chemosaturation in combination with systemic therapy for metastatic uveal melanoma

**DOI:** 10.1002/ijc.70418

**Published:** 2026-03-07

**Authors:** Patrick Kasteleiner, Gerd Grözinger, Jörg Schmehl, Lukas Flatz, Andrea Forschner

**Affiliations:** ^1^ Department of Dermatology Eberhard Karls University of Tuebingen Tuebingen Germany; ^2^ Department of Diagnostic and Interventional Radiology Eberhard Karls University of Tuebingen Tuebingen Germany; ^3^ Center of Radiology, Minimally Invasive Therapies and Nuclear Medicine, SLK‐Kliniken Heilbronn Academic Hospital of Ruprecht‐Karls‐University Heidelberg Heidelberg Germany

**Keywords:** chemosaturation, immunecheckpoint inhibitors, liver metastases, metastatic uveal melanoma, tebentafusp

## Abstract

Uveal melanoma is the most common primary intraocular malignancy in adults. Metastatic disease occurs in approximately 40%–50% of patients, the liver being the predominant site of metastasis (>90%). Treatment options include immune checkpoint inhibitors (ICIs), the bispecific fusion protein tebentafusp, and liver‐directed therapies such as chemosaturation. Despite these available approaches, treatment remains a significant challenge, with a median overall survival of approximately 1 year. In our retrospective study, we investigated the efficacy and tolerability of administering systemic therapy within a defined time interval of ±40 days relative to the date of chemosaturation. Patients included had received either ICI or tebentafusp in close temporal proximity to chemosaturation between November 2023 and July 2024. Treatment efficacy was assessed based on radiologic imaging according to response evaluation criteria in solid tumors, and tolerability was evaluated based on documented adverse events. A total of 10 patients with hepatic metastatic UM could be included in the study. Seven patients received systemic therapy with ICI; three patients were treated with tebentafusp. Hepatic disease control was achieved in all patients. Partial response was observed in 7 of 10 patients, while 3 of 10 patients demonstrated stable disease. Chemosaturation was associated mainly with pancytopenia in 5 of 10 patients. Additionally, immune‐related adverse events of common terminology criteria for adverse events grade I–III were observed. In summary, our findings suggest that administering systemic therapy within the defined interval around chemosaturation can achieve disease control with acceptable tolerability. To further evaluate this therapeutic approach, prospective controlled studies are warranted to assess efficacy and optimize patient safety.

## Graphical abstract

What's new?

Uveal melanoma is the most common primary intraocular cancer in adults, and up to half of patients develop metastatic disease, predominantly in the liver, where prognosis remains poor despite promising treatment options. In this study, the authors evaluated the safety and efficacy of administering systemic therapy within 40 days of liver chemosaturation in a cohort of 10 patients with hepatic metastatic uveal melanoma. Overall, the administration of systemic therapy—either immune checkpoint inhibitors or the immunotherapy tebentafusp—within the defined interval around chemosaturation resulted in disease control in all patients. Toxicities were manageable, supporting further investigation of this combined approach.

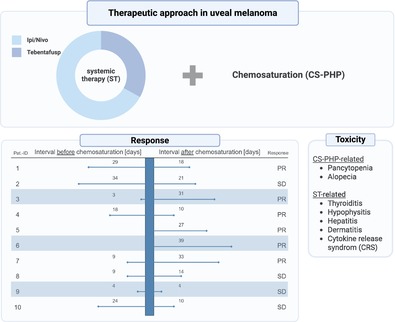

**Abbreviations**
AE: adverse events
CRS: cytokine release syndrome
CS‐PHP: chemosaturation with percutaneous hepatic perfusion
CT: computed tomography
CTCAE: common terminology criteria for adverse events
G‐CSF: granulocyte colony stimulating factor
HLA: human leukocyte antigen
ICI: immune checkpoint inhibitor
irAE: immune‐related adverse events
LDH: Lactate dehydrogenase
MRI: magnetic resonance imaging
OS: overall survival
PD: progressive disease
PFS: progression‐free survival
PR: partial response
SD: stable disease
TMB: tumor mutational burden
UM: uveal melanoma

## 1 INTRODUCTION

Uveal melanoma (UM) is a rare subtype of melanoma arising from melanocytic cells of the uveal tract, which includes the choroid, ciliary body, and iris.^1^ UM represents only approximately 3%–5% of all melanomas, but is, on the other hand, the most common primary intraocular malignancy in adults.^2^ Approximately 40%–50% of patients with UM develop metastases, the liver being the initial site of dissemination in up to 95% of cases.^3^ The development of liver metastases is associated with a poor prognosis and median overall survival (OS) is limited to approximately 1 year.^2^

UM differs significantly from cutaneous melanoma in its underlying molecular pathogenesis, particularly in the spectrum of driver mutations and its characteristically low tumor mutational burden (TMB).^1^, ^2^ The low TMB values are one of the factors that might be associated with the reduced response rates of UM to immune checkpoint inhibitors (ICIs).^4^

Consequently, treatment options of metastasized UM remain challenging. Monotherapy with ICIs such as PD‐1 inhibitors (nivolumab, pembrolizumab) or CTLA‐4 inhibitors (ipilimumab) has demonstrated low objective response rates^5^, ^6^ in real‐world data. However, combined ICI with nivolumab and ipilimumab has shown modest improvements in response rates, progression‐free survival (PFS), and OS in small studies.^3^, ^7^

Since January 2022 (US Food and Drug Administration) and April 2022 (European Medicines Agency), systemic therapy with the bispecific fusion protein tebentafusp has been approved for metastatic UM. Tebentafusp comprises a soluble, affinity‐enhanced T‐cell receptor restricted to human leukocyte antigen (HLA)‐A02:01, specific for glycoprotein 100 (gp100), fused to an anti‐CD3 single‐chain variable fragment. As the first treatment in a clinical phase III trial tebentafusp has demonstrated improved OS and PFS,^8^ however, its utility is constrained by the requirement for HLA‐A02:01 expression, present in only ~45% of patients with UM.

As the liver is the prognostic most relevant organ in metastasized UM, liver‐directed therapies remain an important treatment option in the management of metastatic UM, particularly for patients with non‐resectable liver metastases. Among these, percutaneous hepatic perfusion with melphalan (CS‐PHP) has emerged as a promising locoregional approach.^9^, ^10^ In this procedure, the liver is selectively perfused with high‐dose melphalan via the common hepatic artery, while systemic exposure is minimized through extracorporeal filtration of hepatic venous effluent. A hepatic tumor burden exceeding 50%–60% is generally considered a contraindication to CS‐PHP due to increased risk and reduced efficacy.^11^, ^12^

Given the persistently poor survival outcomes in metastatic UM, there is an urgent need for novel therapeutic strategies. The combination of systemic therapies such as ICI‐based regimens or tebentafusp with liver‐directed interventions like CS‐PHP may offer synergistic benefits. However, data on the concurrent or sequential use of systemic therapy and CS‐PHP remain limited.^13^

This study reports on a retrospective analysis evaluating the efficacy and safety of systemic therapy administered in close time interval to CS‐PHP in patients with metastatic UM.

## 2 PATIENTS AND METHODS

**FIGURE 1:**
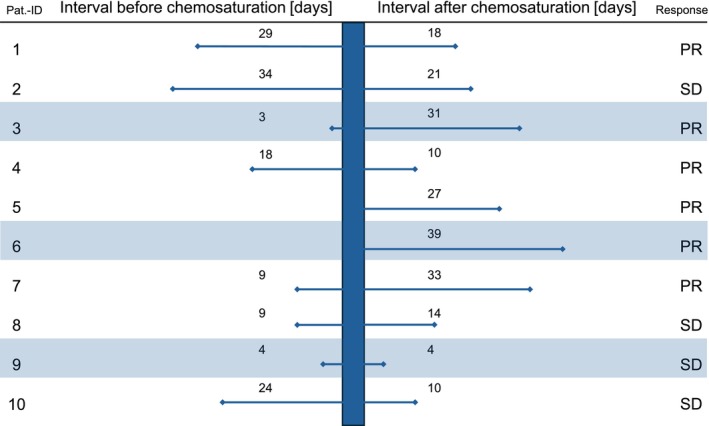
Time interval between systemic therapy and chemosaturation and hepatic response. The time interval between the combined Immune checkpoint inhibition or Tebentafusp (shaded region) and chemosaturation (shown as bar) and the hepatic response (PR = partial response, SD = stable disease) shown for our 10 patients.

A total of 10 patients with metastatic UM were retrospectively analyzed. All patients underwent chemosaturation with percutaneous hepatic perfusion (CS‐PHP) and received systemic therapy with either ICIs or tebentafusp within a time window of ±40 days relative to the CS‐PHP procedure (see Figure 1). Systemic therapy and CS‐PHP were administered due to metastasis of UM, the latter particularly for predominant hepatic metastasis or progression. The study period spanned from November 2023 to July 2024.

Seven patients (*n* = 7) were treated with ICI consisting of ipilimumab (3 mg/kg body weight) and nivolumab (1 mg/kg body weight) every 3 weeks for 4 cycles, followed by nivolumab monotherapy (480 mg every 4 weeks). Three patients (*n* = 3) received tebentafusp at a dose of 68 μg administered weekly. Tebentafusp was administered at 20 μg on day 1, 30 μg on day 8, and 68 μg from day 15 onwards as part of the dose‐escalation regimen.

Two patients (Pat.‐ID 5 and 6) received CS‐PHP prior to systemic therapy, while eight received CS‐PHP after at least one cycle of systemic therapy. One patient (Pat.‐ID 2) received nivolumab after CS‐PHP as part of the ipilimumab and nivolumab regimen.

Relevant criteria for CS‐PHP included a hepatic tumor burden of less than 50%, an Eastern Cooperative Oncology Group performance status of 0 or 1 and no history of heart failure associated with a significantly reduced left ventricular ejection fraction (<50%). All treatment decisions were made by our interdisciplinary tumor board.

Response evaluation was performed about 6 weeks after CS‐PHP had been performed and based on the electronic clinical files, including radiological reports on liver magnetic resonance imaging (MRI), computed tomography (CT) of the neck, thorax, abdomen, and pelvis, and either CT or MRI of the brain. All imaging results were reviewed and discussed within our institutional multidisciplinary tumor board. Tumor response was categorized according to response evaluation criteria in solid tumors criteria as follows: complete response (CR), partial response (PR), stable disease (SD), and progressive disease (PD).

Treatment‐related adverse events (AEs) and safety assessments were assessed by reviewing electronic medical records. Adverse events were documented within a 90‐day window following the date of CS‐PHP.

The patients ranged in age from 40 to 82 years and included both male and female participants with a gender distribution of 1:1 (see Table 1). In addition to hepatic metastases, extrahepatic metastases were present in seven patients (70%). Sites of extrahepatic involvement included lymph nodes, lungs and pleura, bones, and the brain.

**TABLE 1:** Patients characteristics.

	Number of patients *n* (%)
Sex/age (median, range)	
F/(62J., 40–74J.)	5 (50%)
M/(59J., 53–82J.)	5 (50%)
Metastasization	
Hepatic	3 (30%)
Hepatic and extrahepatic	7 (70%)
Systemic therapy	
Ipilimumab/nivolumab	7 (70%)
Tebentafusp	3 (30%)

## 3 RESULTS

### 3.1 Hepatic response

Radiologically, a PR was observed in seven patients (70%). The remaining three patients (30%) had SD. Consequently, disease control of hepatic metastases could be achieved in all patients (10 out of 10, 100%, Table 2 and Figure 1).

### 3.2 Extrahepatic response

Among the seven patients with additional extrahepatic metastases, radiological assessment revealed a PR in one of seven patients (14%). SD and PD were each observed in three of seven patients (43%) respectively. This resulted in an overall extrahepatic disease control rate of 57% in this subgroup (Table 2).

### 3.3 Safety and tolerability

The safety is displayed in Table 2.

Following CS‐PHP, pancytopenia occurred in 5 of 10 patients (50%). In these patients, administration of granulocyte colony‐stimulating factor (G‐CSF) was performed and, in some cases, transfusion of red blood cells and/or platelet concentrates was performed. Additionally, alopecia was reported in one patient (10%). These AEs were considered chemotherapy‐related toxicities induced by melphalan.

Among patients receiving ipilimumab and nivolumab followed by nivolumab monotherapy, typical immune‐related adverse events (irAEs) occurred also after CS‐PHP. These included: autoimmune thyroiditis (one of seven patients), hypophysitis (one of seven patients), immune‐mediated hepatitis common terminology criteria for adverse events (CTCAE) grade III (one of seven patients), dermatitis CTCAE grade II–III (one of seven patients).

During tebentafusp administration, cytokine release syndrome (CRS) of grade I–II occurred in two of three patients (66.6%), all of these during treatment initiation.

### 3.4 LDH as surrogate marker

Lactate dehydrogenase (LDH) levels were assessed as a surrogate marker of tumor burden at or immediately prior to CS‐PHP. Elevated LDH levels (>250 U/L) were observed in 7 of 10 patients (70%), while normal LDH values were found in 3 of 10 patients (30%). Interestingly, in this normal‐LDH group, two patients demonstrated stable hepatic disease (SD), and one patient exhibited a PR.

**TABLE 2:** Response hepatic and extrahepatic, toxicity and LDH levels.

	Number of patients *n* (%)
Response hepatic	
Complete response (CR)	0
Partial response (PR)	7 (70%)
Stable disease (SD)	3 (30%)
Progressive disease (PD)	0
Response extrahepatic	
Complete response (CR)	0
Partial response (PR)	1 (14%)
Stable disease (SD)	3 (43%)
Progressive disease (PD)	3 (43%)
Adverse effects most likely due to chemosaturation	
Pancytopenia	5 (50%)
Alopecia	1 (10%)
Adverse effects most likely due to systemic therapy	
Autoimmune thyroiditis	1
Autoimmune hypophysitis	1
Autoimmune hepatitis (CTCAE grade III)	1
Dermatitis (CTCAE grade II–III)	1
Cytokine release syndrome (CRS) grade I–II	2
LDH levels	
Elevated	7 (70%)
Non‐elevated	3 (30%)

## 4 DISCUSSION

Metastatic UM remains a disease with poor prognosis and limited OS, particularly in cases with hepatic metastases, which are the most significant determinant of outcome. Despite the introduction of new therapeutic options, such as the bispecific fusion protein tebentafusp, prognosis is unfavorable. Although tebentafusp has demonstrated a significantly improved 1‐year OS rate of 73%, the 3‐year OS remains considerably lower at 27%.^8^, ^14^

For the >50% of patients with metastatic UM who are HLA‐A*02:01‐negative and thus ineligible for tebentafusp, treatment currently consists of ICI. In a meta‐analysis by Yamada et al., treatment with ipilimumab and nivolumab yielded a median PFS of 3.0 months, compared to 2.8 months for ICI monotherapy (including pooled results for ipilimumab, nivolumab, pembrolizumab, tremelimumab, and avelumab). Median OS varied substantially, with 16.3 months for dual ICI versus 9.8 months for monotherapy.^15^

A second meta‐analysis by Petzold et al. reported a median OS of 10.9 months for anti‐PD‐1 monotherapy, 7.8 months for anti‐CTLA‐4 monotherapy, and 15.7 months for dual ICI therapy. Corresponding PFS values were 2.7 months for anti‐PD‐1 monotherapy and 3.0 months for both dual ICI and anti‐CTLA‐4 monotherapy.^16^ In summary, these findings highlight a survival benefit with dual ICI,^7^ albeit within the context of modest therapeutic efficacy overall.

Importantly, the efficacy of ICI is known to be reduced in the setting of hepatic metastases compared to non‐hepatic metastases.^17^, ^18^ This may be attributed to reduced T‐cell infiltration and liver‐associated immune tolerance, which fosters T‐cell dysfunction.^19^, ^20^

Percutaneous hepatic perfusion with melphalan (CS‐PHP) is a liver‐directed therapy that offers an effective approach for unresectable hepatic metastases in UM as shown in the FOCUS study, which revealed a median PFS of 9 months and a median OS of 20.5 months following CS‐PHP.^21^ Nevertheless, the prognosis for patients with hepatic metastases remains poor, and the need for innovative therapeutic strategies persists.

In this retrospective study, we evaluated the efficacy and safety of systemic therapy, either tebentafusp or dual ICI, and CS‐PHP in close time intervals of 40 days in a cohort of 10 patients. Our results indicate that disease control of hepatic metastases (PR or SD) was achieved in 100% of patients when CS‐PHP was administered within a ±40‐day interval of systemic therapy. This suggests a potential therapeutic benefit from the combination approach. Importantly, no unexpected safety concerns were identified. Melphalan‐associated pancytopenia^9^, ^11^ was observed in 5 of 10 patients (50%) following CS‐PHP, while irAEs such as autoimmune thyroiditis, hypophysitis, hepatitis (CTCAE grade III), and dermatitis (CTCAE grade II–III) occurred in patients receiving ICIs. Additionally, low‐grade CRS was observed in two of three patients (66.6%) treated with tebentafusp, primarily during initiation.

Our findings are consistent with data from the CHOPIN trial phase Ib on combination of CS‐PHP with ipilimumab and nivolumab.^22^ That study also demonstrated tolerability and excellent disease control. However, in comparison to CS‐PHP alone it reported a higher incidence of CTCAE grade III/IV including febrile neutropenia among others, while grade I/II irAEs were comprising hypothyroidism, hepatitis, and dermatitis.^13^ The increased toxicity might be related to enhanced antigen release from repeated CS‐PHP procedures, potentially triggering heightened immune activation.

The most recently presented data from phase 2 of the CHOPIN trial confirmed excellent disease control and demonstrated a higher incidence of grade III/IV AE as perfusion‐related in the combination cohort, while a smaller percentage experienced grade III/IV AE as ICI‐related. This raises the question if ICI also enhances the toxicity of CS‐PHP. Nevertheless PHP‐related AE were still manageable.

In the CHOPIN trial phase 2, ipilimumab was administered at 1 mg/kg in combination with nivolumab at 3 mg/kg every 3 weeks until week 9. Additionally, chemosaturation was performed in week 1 and 7, between the first and second as well as the third and fourth cycles of combined immunotherapy. In contrast, the intervals in our cohort between the last systemic therapy before CS‐PHP ranged from 3 to 34 days, and from 4 to 39 days between CS‐PHP and the subsequent systemic therapy. While the standardized protocol used in the CHOPIN trial represents an ideal clinical scenario and is certainly desirable, our data demonstrate that a non‐uniform timing between systemic therapy and chemosaturation, reflecting a real‐world setting, can also be therapeutically effective and tolerable in terms of toxicity.

The SCANDIUM II trial compared isolated hepatic perfusion (IHP) followed by four cycles of Ipilimumab 3 mg/kg and Nivolumab 1 mg/kg with one cycle Ipilimumab 3 mg/kg and Nivolumab 1 mg/kg before IHP and continued by three cycles of combined ICI, followed by maintenance of Nivolumab 480 mg for 1 year. The trial reported efficacy for both regimens with an improved disease control in the post‐operative arm, yet not statistically significant. Despite high toxicity from IHP and irAE, the regimens were considered safe. Immune‐related AE were higher in the pre post‐operative arm, which could be attributed to stronger immune activation due to released onco‐antigens during IHP.^23^

Although CS‐PHP is generally considered a safe and effective modality for treating liver metastases in UM,^9^, ^10^ pancytopenia remains a well‐known side effect. In our cohort, pancytopenia occurred in 50% of patients post‐CS‐PHP. While typically manageable, severe cases may pose an increased risk for infection or bleeding. Possible mechanisms include melphalan leakage, either through the perfusion system or due to intrahepatic shunting or neovascularization within metastatic lesions. An increased formation of shunts can be expected with rising tumor burden. Pancytopenia tends to occur within the first 2 weeks post‐procedure. In our patients, G‐CSF was administered after onset of pancytopenia. Subsequently, due to the frequent occurrence of pancytopenias, this approach was adapted to align with the FOCUS study protocol, with G‐CSF being administered within 72 h.^21^ However, considering the recurrence of cytopenias, pegylated filgrastim and closer laboratory monitoring may be warranted in future treatment protocols.

Tebentafusp, administered continuously, demonstrated a favorable safety profile in our cohort. CRS grade I–II occurred during therapy initiation, which aligns with safety data from phase 3 trials and subsequent 3‐year follow‐up analyses.^8^, ^14^ These findings suggest that tebentafusp with CS‐PHP may offer a more tolerable safety profile compared to combinations involving dual ICI.

Nonetheless, ipilimumab and nivolumab carry the risk of immune‐mediated hepatitis, which may delay the administration of CS‐PHP or the continuation of systemic therapy, particularly in HLA‐A*02:01‐negative patients, where tebentafusp is not an option.

Current investigations focus on combining additional local treatment modalities with established systemic therapies. In this context, the combination of stereotactic radiotherapy and immunotherapy has emerged as a potential therapeutic option in cases of limited hepatic metastasis.^24^

In summary, our study demonstrates that the close time interval between tebentafusp or dual ICI with CS‐PHP can help to achieve disease control with acceptable tolerability in patients with metastasized UM. Attention should be given to the management of melphalan‐induced cytopenias and ICI‐associated AEs. Due to the risk of pancytopenia, we recommend laboratory monitoring at least weekly for 1 month following CS‐PHP. In addition, prophylactic administration of G‐CSF, potentially in its pegylated form, should be considered. These encouraging findings warrant further investigation in prospective controlled studies to validate efficacy and optimize safety management in this high‐risk patient population.

## AUTHOR CONTRIBUTIONS

**Patrick Kasteleiner:** Writing – original draft; visualization; data curation; methodology. **Gerd Grözinger:** Data curation; writing – review and editing. **Jörg Schmehl:** Data curation; writing – review and editing. **Lukas Flatz:** Writing – review and editing. **Andrea Forschner:** Writing – review and editing; conceptualization; data curation; methodology; validation; supervision.

## CONFLICT OF INTEREST STATEMENT

Patrick Kasteleiner received speaker's fees from Delcath (institutional). Gerd Grözinger declares no conflict of interest. Jörg Schmehl declares no conflict of interest. Lukas Flatz received grants from Hookipa Pharma, Swiss Cancer League, German Research Foundation, Immunophotonics, Mundipharma. Lukas Flatz received consulting fees from Philogen and support for attending meetings or travel from Philogen, Hookipa Pharma. Lukas Flatz participates on board for the University of Basel (TIL trial, unpaid) and is founder of Hookipa Pharma, Schmelzberg, Humion, and Abtherix—all outside the submitted work. Andrea Forschner received travel support and/or speaker's fees and/or advisor's honoraria by Novartis, BMS, MSD, Pierre Fabre, Delcath, Immunocore and research funding from Stiftung BMS Immunonkologie outside the submitted work.

## ETHICS STATEMENT

All patients included in this study provided written informed consent for documentation of their clinical data for research purposes and publications. The local ethics committee of the medical faculty of the University Tuebingen has approved the research project (No. 445/2025BO2). This study has been performed in accordance with the general recommendations outlined in the Declaration of Helsinki.

The authors of this manuscript declare that in the writing process of this work, no generative artificial intelligence (AI) or AI‐assisted technologies were used to generate content, ideas, theories, images or graphical elements. During the preparation of this work, the authors used ChatGPT in order to enhance readability and refine language. After using this tool, the authors carefully reviewed and edited the manuscript to ensure its accuracy and coherence. The authors take full responsibility for the content of the publication.

## Data Availability

The authors confirm that the data supporting the findings of this study are available on reasonable request from the corresponding author.
